# Laparoscopic transgastric resection of a gastric leiomyosarcoma: report of a rare case

**DOI:** 10.3332/ecancer.2022.1419

**Published:** 2022-07-04

**Authors:** Nicolas Panzardi, Matias Mihura-Irribarra, Daniela Speisky, Joaquin Fernandez-Alberti, Mariana Toffolo Pasquini, Pablo Dezanzo, Daniel Enrique Pirchi

**Affiliations:** 1 General Surgery Department, Hospital Británico de Buenos Aires, Perdriel 74, C1280 AEB Buenos Aires, Argentina; 2 Esophageal and Gastric Surgery Department, Hospital Británico de Buenos Aires, Perdriel 74, C1280 AEB Buenos Aires, Argentina; 3 Pathology Department, Hospital Británico de Buenos Aires,Perdriel 74, C1280 AEB Buenos Aires, Argentina; a https://orcid.org/0000-0002-1092-1132; b https://orcid.org/0000-0001-7131-7951; c https://orcid.org/0000-0002-5237-8924; d https://orcid.org/0000-0002-1827-7361; e https://orcid.org/0000-0001-9893-2487; f https://orcid.org/0000-0002-7353-0470

**Keywords:** gastric leiomyosarcoma, laparoscopic, transgastric resection, case report

## Abstract

Gastric leiomyosarcoma is a rare type of tumour that is far less prevalent than gastrointestinal stromal tumours. We describe a case of a 42-year-old male patient who consulted for upper abdominal pain. Blood work revealed low haemoglobin levels, requiring red blood cell transfusions. An esophagogastroduodenoscopy was performed, showing a submucosal tumour with central ulceration in the greater gastric curvature. The patient underwent an endoscopic ultrasound with fine needle biopsy and the sample showed a spindle cell neoplasia. Computed tomography scan demonstrated absence of distant metastases. Upon multidisciplinary consensus, it was decided to perform surgery. A laparoscopic approach was conducted, where no peritoneal lesions were observed. Transgastric resection of the tumour was performed. Free tumour margins were achieved following oncologic criteria (minimum tumour manipulation and one-piece resection without damaging the tumour capsule). After exhaustive sampling, the final pathology report informed an 11 × 9 × 5 cm gastric leiomyosarcoma. Immunohistochemical examination showed positivity with smooth muscle actin, muscle-specific actin, calponin and desmin. The patient had an uneventful recovery, and 6 post-operative months’ clinical, tomographic and endoscopic control informed no disease recurrence. To the best of our knowledge, there are less than 20 published cases of patients with diagnosis of gastric leiomyosarcoma. This study highlights the importance of reporting this entity, in order to contribute to the available literature concerning this topic.

## Introduction

Leiomyosarcomas (LMS) are a type of sarcoma originating from smooth muscle cells, which have the ability to grow almost anywhere in the human body. LMS of the gastrointestinal (GI) tract are rare neoplasms and are far less prevalent than other mesenchymal tumours, such as GI stromal tumours (GISTs), which, although they originate from the cells of Cajal, have similar macroscopic and microscopic features. GISTs and LMS were misdiagnosed until early 2000s, when GISTs became recognised as a distinct entity [1]. The main difference between both tumours lies on the expression of the KIT receptor (CD117), present in almost 95% of GISTs, as well as the DOG-1 marker, expressed in most of the CD117-negative GISTs. Another representative immunohistochemical marker is CD34, that even though is not specific for GISTs, is seen in up to 60–70% of these kind of tumours [2]. LMSs are usually negative for KIT and DOG-1 markers and show positivity for smooth muscle actin (SMA), desmin, calponin, H-caldesmon and smoothelin [3].

The importance in the difference between these two entities relies on the response that GISTs show to KIT-directed therapy, such as Imatinib or Sunitinib, and also their different prognosis.

The aim of this article is to present a case of a rarely encountered tumour, contributing to the international literature.

## Case presentation

On July 2021, a 42-year-old man consulted our hospital referring upper abdominal pain associated to asthenia for the last 4 days. His past medical history revealed grade II obesity (body mass index = 38.5). Physical examination evidenced mild epigastric tenderness and a non-complicated umbilical hernia, but was otherwise unremarkable. Blood work revealed a haemoglobin value of 6.6 mg/dl, requiring transfusions of two red blood cell units.

The patient underwent an esophagogastroduodenoscopy, finding an 80 mm submucosal tumour with central ulceration located along the greater curvature and anterior wall of the upper gastric body. The biopsy revealed superficial mucosal erosions without atypical cells.

Thereafter, and upper endoscopic ultrasound (EUS) was performed, evidencing a hypoecoic lesion of 60 × 58 mm with well-defined borders (Figure 2a and b). This lesion involved up to the gastric serosa. Fine needle biopsy (FNB) was performed and sent to pathology. Histological sections of the material embedded in paraffin and coloured with haematoxylin and eosin showed bundles of well-differentiated spindle cells with blunt-ended nuclei and eosinophilic cytoplasm. Mitotic figures were not seen in this sample. Immunohistochemical techniques were carried out on 3-micron histological samples using an automated system in accordance with the manufacturer’s guidelines (Benchmark XT, Ventana). Diffuse positivity was observed with SMA, muscle-specific actin, calponin and desmin. On the contrary, no reactivity was demonstrated for CD117, DOG-1, CD34, β-catenin, and S-100. Ki67 proliferation index in this sample was around 8%. All these findings were in accordance with a spindle cell tumour with smooth muscle differentiation (Figure 3).

**Figure 1.**
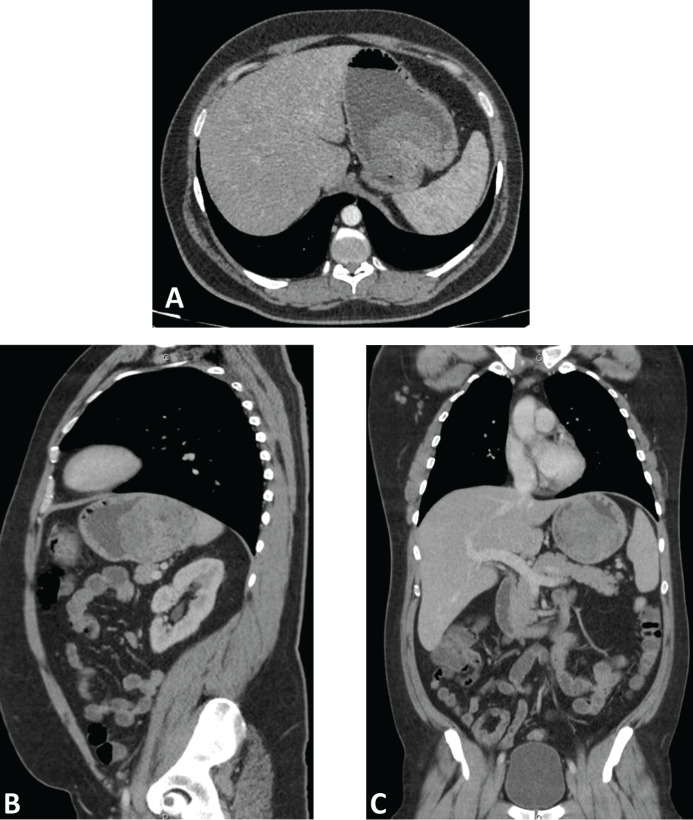
Computed tomography with intravenous and oral contrast. Axial (A), parasagital (B) and coronal (C) planes showing a highly-vascularized tumor with an endophytic growth in the upper gastric body measuring 90 x 89 x 70 mm.

**Figure 2.**
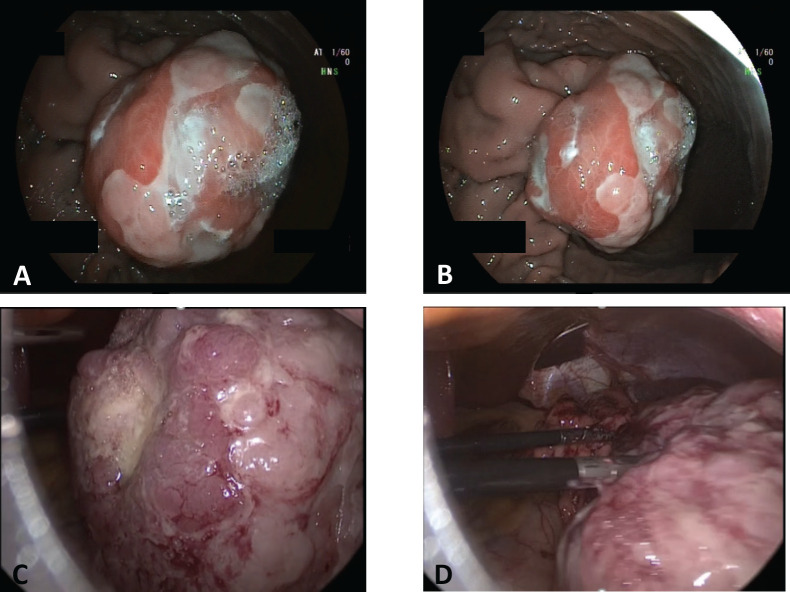
Gastric tumor. Endoscopic and laparoscopic view. A & B) Endoscopic view of the submucosal tumor located in the greater curvature of the upper gastric body. C & D) Laparoscopic view of the tumor after performing the gastrotomy.

A computed tomography (CT) scan was performed, showing a highly vascularised tumour with an endophytic growth in the upper gastric body measuring 90 × 89 × 70 mm (Figure 1). No distant metastases were seen.

After multidisciplinary consensus, a surgical resection was decided, performing a laparoscopic transgastric resection with minimal tumour manipulation.

We began with a diagnostic laparoscopy in which no distant metastases were seen and multiorgan invasion was ruled out. Because of the predominant endophytic growth of the tumour, we performed an intraoperative EGD in order to assess tumour-free resection margins and make sure an R0 resection was feasible. Under maximum gastric insufflation, free margins were identified and thus we continued with the resection. Gastrostomy across the non-pathological gastric wall was performed with ultrasonic shears achieving at least a 1 cm margin along the whole tumour circumference. Complete resection with tumour-free margins was accomplished. Gastrostomy was closed with a combined technique of stapled 60 mm sutures and manual continuous suturing with an absorbable material. Hydropneumatic test was performed during endoscopy without evidencing any leakage. The specimen was retrieved in an endobag through the patient’s umbilical hernia which was opened up to a total length of 10 cm. The patient had an uneventful recovery and was discharged home on the third post-operative day.

**Figure 3.**
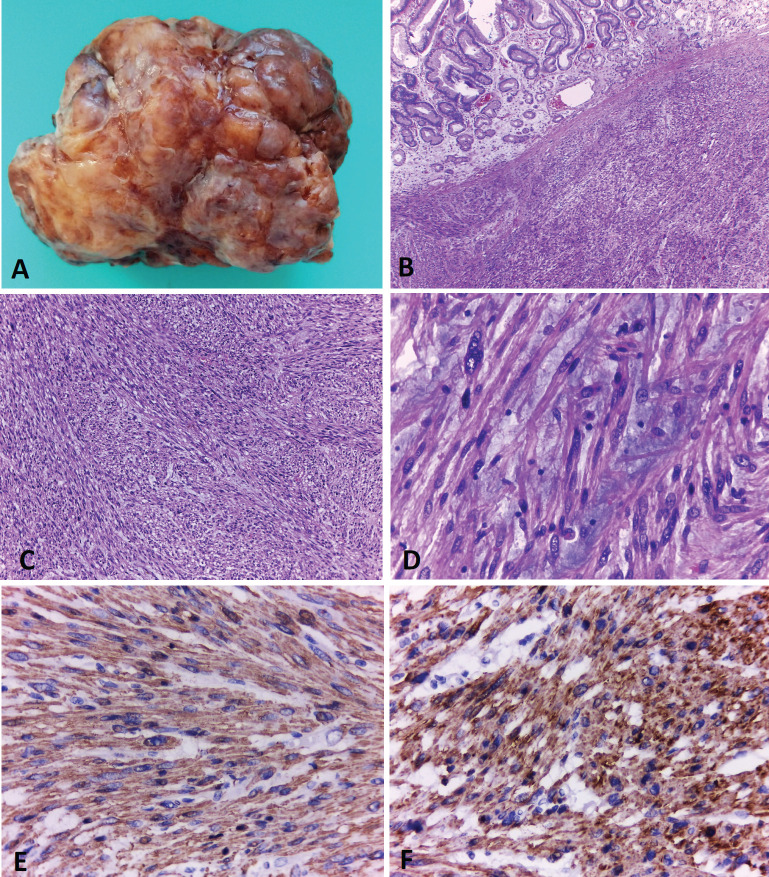
Gastric leiomyosarcoma, Macroscopic and microscopic examination. A) Surgical specimen of a solid polypoid mass measuring 11 × 9 cm. B) Histological sections (H&E) of normal gastric mucosa with underlying neoplastic proliferation of spindle cells. C). The tumour is composed of interlacing fascicles of spindle cells, with elongated blunt-ended nuclei and eosinophilic cytoplasms. D) Some areas of the neoplasia showed marked atypia and a myxoid background. E and F) Immunohistochemical techniques. Positivity with smooth muscle actin (E) and specific muscle actin (F). Original magnification: 40x (B); 100x (C); 400x (D-F).

Macroscopic examination of the specimen showed an 11 x 9 x 5 cm solid endophytic mass covered with eroded mucosa compromising the gastric wall (Figure 3a). Exhaustive sampling of the tumour was performed. Surgical margins were tumour-free.

Microscopic examination showed neoplastic proliferation of spindle cells arranged in fascicles, some of them marked with atypia and areas of myxoid background (Figure 3). The neoplasm involved the mucosa, submucosa and muscular gastric layers. In these samples, 20 mythotic figures per 10 HPF were counted and Ki67 proliferation index was close to 30%. The immunohistochemistry findings of the surgically resected tumour were in line with the EUS–FNB findings and the patient was finally diagnosed with a gastric leiomyosarcoma.

This case was discussed in the multidisciplinary tumour board and follow-up without adjuvant therapy was decided. After 6 months of post-operative follow-up, the patient remains free of disease, with unremarkable CT scan and endoscopic control.

## Discussion

We present a rare case of a gastric leiomyosarcoma in a 42-year-old man resected with a laparoscopic transgastric approach. To the best of our knowledge, there are less than 20 cases reported worldwide [4].

LMSs of the GI tract are rare entities, with gastric localisation being one of the least common sites. The most frequent clinical presentations consist of obstruction, perforation, pain or subclinical bleeding. Although there has been a case of massive haematemesis reported [5], massive bleeding due to gastric LMS is infrequent, with very few reported cases.

EGD usually reveals submucosal tumours with normal or ulcerated mucosa. EUS has been proved to be the best diagnostic tool in this type of tumours, with a sensitivity close to 97% [6]. Biopsy is useful in order to differentiate LMS from GISTs or other subepithelial tumours, such as leiomyomas.

Immunohistochemistry is the key to achieve a definitive diagnosis. GISTs present pathognomonic findings, such as KIT positivity and DOG-1 immunoreactivity, even in rare KIT-negative GISTs [7]. These two markers possess a sensitivity for the diagnosis up to 94.7%. LMS, on the other hand, are KIT- and DOG-1-negative, and show high positivity for SMA, desmin, calponin, h-caldesmon and smoothelin [8]. In our case, EUS with fine needle aspiration (FNA) was useful to establish the smooth muscle differentiation of the neoplasia, although the final diagnosis of LMS was confirmed with the surgical specimen.

The type of surgery depends on the size and localisation of the tumour, ranging from limited organ-preserving procedures (such as wedge gastrectomies or endoscopic resections) to major surgeries, such as total gastrectomies or multiorganic resections [9]. The only endoscopic resection of an LMS published was reported by Sato *et al* [10] in a patient with a 23-mm tumour that was excised by a submucosal dissection technique. In our case, we could accomplish oncology criteria (minimum manipulation, free tumour margins and one-piece resection, without damaging the tumour capsule) by a laparoscopic approach. There are multiple well-known advantages of laparoscopy, such as shorter hospital stay, lower risk of wound complications, less post-operative pain and faster return to daily activities. Therefore, if oncological criteria can be assured, laparoscopic resection is the best choice in large volume centres with experience in minimally invasive procedures.

Surgical resections with organ preservation vary with many different options. The most common ones are wedge gastrectomies and transgastric resections. Wedge gastrectomies are usually preferred in tumours located in the greater curvature with an exophytic growth, while transgastric resections are preferred in tumours located close to the EG junction, or those with a predominantly endophytic growth. The reason for this is the difficulty in ensuring an R0 resection of an endophytic tumour with a closed wedge resection, and with this technique we can preserve more normal gastric tissue [11].

It has been demonstrated that tumour-free resection margins and tumour size directly affect the patient’s prognosis [12], and that is why frozen biopsy should be conducted when free margins cannot be confirmed macroscopically during surgery. In our case, since we had a preoperative biopsy performed, and we also assessed gross free-tumour margins intraoperatively by performing laparoscopic and endoscopic combined surgery, we considered that frozen biopsy was not needed.

Concerning systematic lymphadenectomy, it has not been proved to have clear benefits for patient survival, given that LMSs have predilection for haematogenous spread [3]. In our case, laparoscopic transgastric resection with tumour-free margins was performed without lymphadenectomy.

The overall median survival reported for primary GI LMS is 32 months and the estimated 5-year survival is about 22% [12]. The prognostic factors that have been associated with worse outcomes are tumour grade, size of the LMS and resection status. The patient age, gender and signs or symptoms did not show correlation with prognosis [12]. Our patient was studied with CT scan and upper endoscopy at 6 months after surgery and remains free of recurrence.

## Conclusion

Gastrointestinal LMSs are rare malignant neoplasms which must be differentiated from GISTs and other spindle cell tumours in relation to their different prognosis and response to targeted therapies. To the best of our knowledge, there have been less than 20 reported cases of this entity. We present the case of a young patient with a gastric LMS suspected by EUS–FNB and confirmed by surgical specimen samples, who was successfully treated by a laparoscopic transgastric approach with optimal accomplishment of the oncological principles.

## Conflicts of interest

None.

## Funding

No source of funding.
